# Cockayne Syndrome: The many challenges and approaches to understand a multifaceted disease

**DOI:** 10.1590/1678-4685-GMB-2019-0085

**Published:** 2020-05-20

**Authors:** Alexandre Teixeira Vessoni, Camila Chaves Coelho Guerra, Gustavo Satoru Kajitani, Livia Luz Souza Nascimento, Camila Carrião Machado Garcia

**Affiliations:** 1 Washington University School of Medicine, Saint Louis, MO, USA.; 2 Universidade Federal de Ouro Preto, Instituto de Ciências Exatas e Biológicas, Núcleo de Pesquisa em Ciências Biológicas & Departamento de Ciências Biológicas, Ouro Preto, MG, Brazil.; 3 Universidade de São Paulo, Instituto de Ciências Biomédicas, Departamento de Microbiologia, São Paulo,SP, Brazil.

**Keywords:** Cockayne syndrome, transcription-coupled nucleotide excision repair, neurodegeneration, progeroid syndrome, DNA repair

## Abstract

The striking and complex phenotype of Cockayne syndrome (CS) patients combines progeria-like features with developmental deficits. Since the establishment of the *in vitro* culture of skin fibroblasts derived from patients with CS in the 1970s, significant progress has been made in the understanding of the genetic alterations associated with the disease and their impact on molecular, cellular, and organismal functions. In this review, we provide a historic perspective on the research into CS by revisiting seminal papers in this field. We highlighted the great contributions of several researchers in the last decades, ranging from the cloning and characterization of CS genes to the molecular dissection of their roles in DNA repair, transcription, redox processes and metabolism control. We also provide a detailed description of all pathological mutations in genes *ERCC6* and *ERCC8* reported to date and their impact on CS-related proteins. Finally, we review the contributions (and limitations) of many genetic animal models to the study of CS and how cutting-edge technologies, such as cell reprogramming and state-of-the-art genome editing, are helping us to address unanswered questions.

## The epidemiology of Cockayne Syndrome (CS) and the discovery of the CS genes

Edward Alfred Cockayne first described CS in 1936. He diagnosed it in two young siblings (born to healthy parents) that displayed a set of very similar characteristics that included skin photosensitivity, short stature, prominent superior maxillae, disproportionally large hands and feet, sunken eyes with retinal atrophy, hearing impairment, below-average intelligence, a severely limited speaking ability, and muscle contraction, conditions that would later become characteristic of CS patients (Cockayne, 1936, 1946; Laugel, 2013). Subsequent reports also confirmed that patients with CS feature complex and heterogeneous neuropathology that includes calcification of the basal ganglia, cerebellar atrophy, loss of Purkinje and granular cells, hyperchromatic macroglial cells, microcephaly, and patchy demyelination (Guardiola *et al.*, 1999; Karam *et al.*, 2000; Weidenheim *et al.*, 2009; Wilson *et al.*, 2015; Karikkineth *et al.*, 2017).

CS is an autosomal recessive disorder with a prevalence of ~2.7 per million births in Western Europe and in Japan (Kleijer *et al.*, 2008; Kubota *et al.*, 2015). The phenotype of the patients can range from mild to very severe and is subdivided into three types. The classical type (type I) corresponds to the moderate phenotype in which life expectancy is 16 years. In type II (the most severe and with the earliest onset), life expectancy is 5 years, whereas in the third type (mild and atypical), the phenotype manifests itself later in life, with life expectancy above 30 years. In all cases, pneumonia/respiratory ailments are the most common causes of death (Natale, 2010).

*In vitro* culture culture of skin fibroblasts derived from patients with CS in the 1970s was the first step toward the development of experimental models of the disease. CS fibroblasts are characterized by extreme sensitivity to ultraviolet light (UV) despite a normal ability to excise pyrimidine-dimers from the genome (Schmickel *et al.*, 1977; Andrews *et al.*, 1978). In fact, CS cells display a marked defect in the recovery of RNA synthesis after UV irradiation (Mayne and Lehmann, 1982) owing to a failure in the repair of transcriptionally active genes (Venema *et al.*, 1990; van Hoffen *et al.*, 1993). By evaluation of post-UV RNA synthesis recovery in multinucleated cells obtained by the fusion of cells from different CS donors, three complementation groups (A, B, and C) were initially identified (Tanaka *et al.*, 1981; Lehmann, 1982). Group C identified by Lehmann corresponded to a patient that had combined features of CS and *xeroderma pigmentosum* (XP). Patients that fall in this category (termed XP/CS) manifest, in addition to CS features, the classical XP characteristics (skin pigmentation and extremely high skin cancer predisposition) and harbor mutations in the genes *XPB*, *XPD*, *XPG*, or (more recently identified) *XPF* or *ERCC1* (Weeda *et al.*, 1990; Kashiyama *et al.*, 2013; Lehmann *et al.*, 2014; Moriel-Carretero *et al.*, 2015).

In the 1990s, the genes corresponding to groups A and B were cloned, characterized, and termed *CSA* and *CSB*, respectively. *CSB* was originally termed *ERCC6* (excision repair cross-complementation group 6) because it was found to complement the nucleotide excision repair (NER) defect of the Chinese Hamster Ovary mutant cell line UV61, a representative of complementation group 6 of rodent cell lines defective in excision repair (Troelstra *et al.*, 1990). Two years later, Hoeijmaker’s group demonstrated that the expression of this gene could reverse UV sensitivity and rescue post-UV RNA synthesis in a cell line (CS1AN) assigned to CS group B but not in group A cells (Troelstra *et al.*, 1992). Another two years later, using episomal vectors to drive the expression of a cDNA library, Friedberg’s group was able to identify the gene capable of reversing the UV sensitivity of CS cells from group A (but not B) and to reactivate the expression of a UV-inactivated reporter gene (Henning *et al.*, 1995). They discovered that the *CSA* protein, encoded by *ERCC8* (Itoh *et al.*, 1996), can interact with the CSB protein. Both genes play a critical role in the transcription-coupled nucleotide excision repair (TC-NER) of damaged DNA, which is described below in more detail.

## CSA and CSB in TC-NER

**Figure 1:**
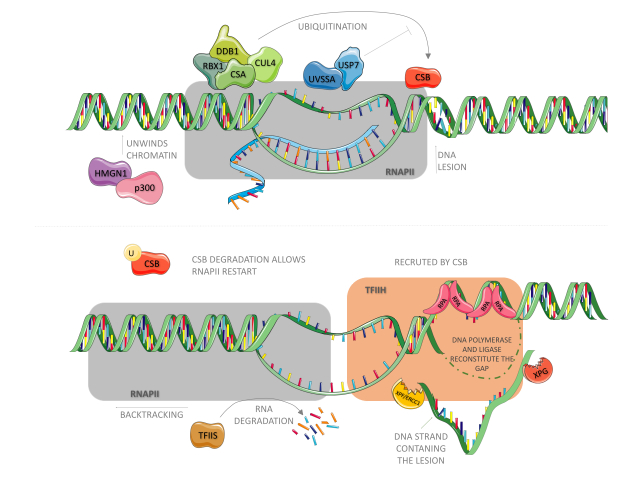
Physical blockage of RNA polymerase II facilitates CSB binding to it. The following is responsible for recruiting p300, CSA and the other NER factors. CSA, together with CUL4, RBX1 and DDB1 are constantly ubiquitinating CSB, however UVSSA-USP7 complex are constantly removing ubiquitin tags from it. CSA also recruits HMGN1, which together with p300 unwinds chromatin upstream RNApol II, allowing it to backtrack and expose the lesion site to NER factors. TFIIS stimulates RNA cleavage by RNApol II during this process. NER factors unwinds DNA around the lesion. While RPA protects ssDNA from degradation, XPG and XPF-ERCC1 endonucleases cleave the strand containing the lesion. DNA polymerase and ligase then fill up the gap. CSB degradation is necessary to RNA synthesis recovery.

Cells evolved complex and refined mechanisms to prevent genome instability in response to the presence of exogenously and endogenously generated DNA lesions. One of these mechanisms, conserved from bacteria to humans, is the NER pathway (Schärer, 2013; Marteijn *et al.*, 2014; Gregersen and Svejstrup, 2018). This system drives the repair of bulky distorting DNA lesions (such as those induced by UV and by some redox processes) in four sequential steps: i) detection of a lesion, ii) excision of a DNA single-strand fragment containing the lesion, iii) DNA synthesis by a polymerase to fill the gap, and iv) nick sealing by a ligase (Costa *et al.*, 2003; Reardon and Sancar, 2005; Menck and Munford, 2014). In bacteria, three proteins (UvrA, UvrB, and UvrC) are critical for the detection and excision of the lesion (Seeberg and Strike, 1976 ; Sancar and Rupp, 1983), whereas in humans, more than 30 proteins acting in an orchestrated manner are required for these steps, as reviewed elsewhere in detail (Menck and Munford, 2014). NER is subdivided into two sub-pathways: global genome repair (GG-NER) and transcription-coupled repair (TC-NER). They differ in how DNA lesions are detected, although the excision and DNA re-synthesis steps are shared by the two pathways (Menck and Munford, 2014). In humans, GG-NER is initiated by the XPC protein (Sugasawa *et al.*, 1998), which is constantly scanning the whole genome for the presence of helix-distorting lesions (Hoogstraten *et al.*, 2008), and the detection of UV products is facilitated by XPE/DDB2 (Cleaver *et al.*, 2009). In TC-NER, as shown in Figure 1, the triggering event is the arrest of RNA polymerase II (RNA pol II) owing to the presence of a lesion in the actively transcribed strand of a gene (Bohr *et al.*, 1985; Hanawalt and Spivak, 2008). It is in this pathway that the CSA and CSB proteins play a critical part. Upon RNA pol II blockade, the binding of CSB to RNA pol II is stabilized (Tantin *et al.*, 1997; van den Boom *et al.*, 2004; Fousteri *et al.*, 2006) and CSB then wraps DNA around itself, altering its conformation and recruiting histone acetyltransferase p300 and core NER factors (such as RPA, XPG, and TFIIH) to RNA pol II arrest sites (Fousteri *et al.*, 2006), as presented in Figure 1 (upper panel). CSB also recruits an E3-ubiquitin ligase complex, highlighted in green, composed of CSA (which contains WD motifs involved in protein-protein interactions), DDB1, Cullin 4A, and ROC1/Rbx1 proteins (Groisman *et al.*, 2003; Fousteri *et al.*, 2006). Although CSA is not necessary for the recruitment of NER factors, it is required for recruiting HMGN1, XAB2, and TFIIS to RNA pol II arrest sites (Fousteri *et al.*, 2006). XAB2 is an XPA-interacting protein (Nakatsu *et al.*, 2000) and might act as a scaffolding factor for protein assembly during TC-NER (Fousteri and Mullenders, 2008), while the nucleosome-binding protein, HMGN1, was suggested to promote chromatin changes that allow for the incision step (Fousteri and Mullenders, 2008).

The fates of the nascent transcript and that of the stalled RNA pol II are still debated. One hypothesis is that transcript cleavage may occur in an elongation factor for RNA pol II (ELL)-dependent manner (Gregersen and Svejstrup, 2018). As for the stalled RNA pol II — which occupies a space that ranges from 25 nucleotides downstream of the lesion to 10 nucleotides upstream (Spivak and Ganesan, 2014), thus impairing the assembly of NER factors — it may undergo reverse translocation/backtracking (Donahue *et al.*, 1994; Fousteri *et al.*, 2006) or be targeted for proteasomal degradation by ubiquitination (Harreman *et al.*, 2009), as shown in Figure 1 (lower panel).

Upon lesion resolution, the CSA-E3 ubiquitin ligase complex performs a critical function in the recovery of transcription by targeting CSB for proteasomal degradation (Groisman *et al.*, 2006). To avoid premature degradation of CSB, UVSSA, which binds firmly to stalled RNA pol II, recruits USP7, an enzyme that promotes deubiquitination of CSB (Schwertman *et al.*, 2012), highlighted in blue in Figure 1. Therefore, once the DNA lesion is removed, USP7-mediated deubiquitination of CSB ceases, and CSB is finally targeted for degradation.

In the absence of the CSA or CSB protein, the arrest of RNA pol II persists, an event that leads to p53 activation and cell death, thus explaining the extreme sensitivity of CS cells to UV damage (Ljungman and Zhang, 1996). Besides its participation in TC-NER, proteins CSA and CSB play several other important roles. In the following sections, we provide detailed descriptions of the structures of these proteins, their functions outside of TC-NER, and a comprehensive review of pathological mutations, their consequences for protein function, and their association with patients’ clinical characteristics.

## The structure and functions of proteins CSA and CSB, or how pathological mutations are (not) associated with clinical phenotypes

CSB is a 168 kDa protein composed of 1493 amino acid residues and is encoded by the *ERCC6* gene located in chromosomal region 10q11 (Troelstra *et al.*, 1992, 1993). It belongs to the SWI2/SNF2 family of helicases, and just as all the proteins in this family, it does not have the capacity to open the DNA double helix (Selby and Sancar, 1997a). In contrast, the SWI2/SNF2 proteins temporarily modify DNA conformation via ATP hydrolysis, thereby altering the DNA contact with histones and nucleosome positioning. Therefore, SWI2/SNF2 proteins are considered chromatin remodelers (Lusser and Kadonaga, 2003; Beerens *et al.*, 2005). In addition to its known classic function in TC-NER, CSB takes part in the regulation of transcription and assists with nuclear and mitochondrial base excision repair (BER). It has been reported that CSB interacts with proteins XPB, XPD, XPG, TFIIH, RNA pol I and II, and glycosylases (Tantin, 1998; Bradsher *et al.*, 2002; Tuo *et al.*, 2002; Sarker *et al.*, 2005; Stevnsner *et al.*, 2008; Kamenisch *et al.*, 2010), although some of these interactions were described only once and need additional confirmation of their biological relevance.

The CSB structure mainly includes the following domains: an acidic domain, SNF2/ATPase region, ubiquitin-binding domain, and a nuclear localization signal (Liu *et al.*, 2015). The acidic domain comprising amino acid residues 356 to 394 is located in the N-terminal portion, which is mostly negatively charged (Troelstra *et al.*, 1992). In other proteins, this domain facilitates protein-protein interactions, especially those of nuclear and DNA-binding proteins, such as transcriptional activators and chromatin remodelers (Melcher, 2000; Carpenter *et al.*, 2005; Wu *et al.*, 2017). Given that CSB is classified as a chromatin remodeler, it has been hypothesized that its acidic domain facilitate this activity, but the possible underlying mechanisms have not yet been fully elucidated (Brosh *et al.*, 1999).

**Figure 2:**
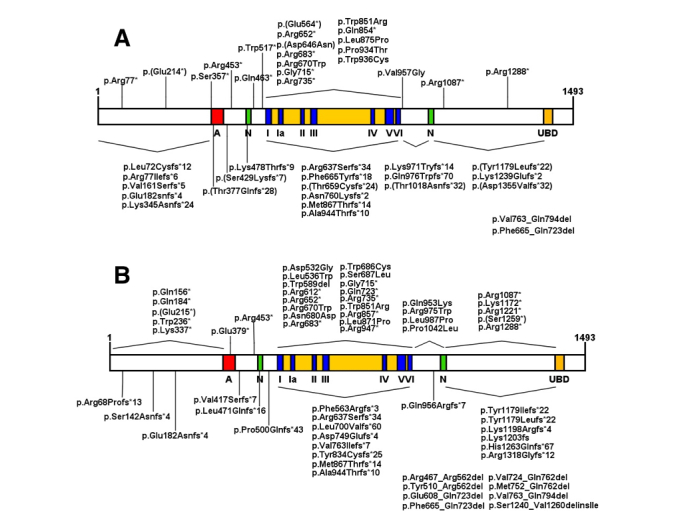
Representation of CSB protein and domains. Acidic region (A), nuclear localization signal (N), helicase motifs (I, Ia -VI) and ubiquitin binding domain (UBD). (A) Homozygous mutations are indicated: frameshifts and nonsense mutations are indicated above the protein, while deletions and missense mutations are indicated below the protein. (B) Represents heterozygous mutations.

Mutations in the acidic domain of CSB in UV61 cells do not compromise the ability to repair lesions caused by UV, 4-QNO (4-Nitroquinoline 1-oxide), and NA-AAF (*N*-acetoxy-2-acetylaminofluorene), or cell viability after exposure to these agents (Brosh *et al.*, 1999; Sunesen *et al.*, 2000). These data indicate that the integrity of this domain is not essential for this protein’s function in TC-NER. Similar results were obtained by Lake *et al.* (2010) with UV irradiation of CS1AN-SV cells expressing CSB protein lacking the first 454 amino acid residues in the N-terminal portion, demonstrating that the absence of this region does not compromise the ability of the protein to associate with chromatin but instead makes such associations much more frequent even without UV exposure. It was also observed that this deletion increases the ATPase activity of CSB, indicating that the N-terminal portion acts as a negative regulator of its association with chromatin via ATP hydrolysis (Lake *et al.* (2010)). CSB protein structure and homozygous and heterozygous pathological alterations are illustrated in Figure 2 A and B, whereas Table S1 lists all ERCC6 mutations reported in the literature.

The nuclear localization signal is found within regions 466–481 and 1038–1055 (amino acid positions) (Lange *et al.*, 2007). In a recent work, Iyama *et al.* (2018) identified through computational analysis the existence of a third region of nuclear localization signal, in addition to the three nucleolar localization signals that cooperate for the distribution of the protein between the nucleus and nucleolus.

Among these regions there is also the SNF2/ATPase domain, which is highly conserved in the SWI2/SNF2 family (Pazin and Kadonaga, 1997). This domain extends from amino acid residue 510 to residue 960 and contains seven ATPase motifs: I, Ia, II, II, IV, V, and VI (Troelstra *et al.*, 1992), essential for the functioning of the protein (Brosh *et al.*, 1999; Selzer *et al.*, 2002). The function of the ATPase region is the most relevant for the activity of CSB, because this function provides energy for its association with (and remodeling of) chromatin by altering the positioning of nucleosomes (Citterio *et al.*, 2000). Through this activity, CSB enables the repair of DNA lesions by promoting the access of other proteins, such as CSA and NER factors, to the site of stalled RNA pol II (Stadler and Richly, 2017). During the transcription process, CSB alters *in situ* chromatin conformation, favoring the binding of transcription factors (Lake *et al.*, 2014).

To understand and characterize the functional importance of each ATPase motif, several cell lines carrying mutations in different and highly conserved regions in these motifs have been created. In general, amino acid substitutions in these regions decrease cell survival, RNA synthesis recovery, and DNA repair after exposure to UV, as well as increase the sensitivity to 4-NQO (Brosh *et al.*, 1999; Muftuoglu *et al.*, 2002; Selzer *et al.*, 2002). Notably, mutations in domains I and II, named “Walker A” and “Walker B,” respectively, can completely inactivate the ATPase activity (Citterio *et al.*, 1998; Christiansen *et al.*, 2003). Mutations in motifs V and VI also compromise the ATPase activity, although to a lesser extent (Christiansen *et al.*, 2003). Different motifs can also contribute in different ways to other activities carried out by CSB. Tuo *et al.* (2001) demonstrated that cells mutated in motifs V and VI are more sensitive to γ-radiation than wildtype cells, and DNA lesions such as 7,8-dyhydro-2’-deo-xyguanosine (8-oxoGua) accumulate in CSB-null and VI mutant-CSB cells after exposure to γ-radiation, indicating a possible relation between CSB and the BER pathway (Tuo *et al.*, 2002a).

The ubiquitin-binding domain (UBD) is located in the C-terminal region of the CSB protein (amino acid residues 1400–1428). UBD-CSB-deficient cells have a phenotype similar to that of cells that do not express the CSB protein at all. Although the TC-NER complex is fully assembled around the lesion and RNA pol II in these cells, the repair does not proceed because of the inability of CSB to leave the lesion site (Anindya *et al.*, 2010). The replacement of the CSB UBD by another UBD, such as UBA2 of Rad23, an otherwise unrelated *Saccharomyces cerevisiae* DNA repair gene, also enables CSB dissociation from the lesion region and progression of the repair process, thereby demonstrating the need for CSB ubiquitination for the correct functioning of the protein in this TC-NER (Anindya *et al.*, 2010). Cells lacking UBD in the CSB protein are sensitive to oxidatively induced DNA damage (Ranes *et al.*, 2016), suggesting that this domain is important for the repair of this kind of lesion. The conserved amino acid lysine at position 911 was recently found to be a ubiquitination site that is also required for this function, but is dispensable for TC-NER (Ranes *et al.*, 2016).

By constructing several CSB mutants with different deletions in the C-terminal region, Sin *et al.* (2016) found that the integrity of the amino acid sequence in this region is important for this sumoylation of this protein and association with chromatin. Aside from this region, a functional UBD domain is necessary for RNA Pol II interaction and CSA recruitment to the nucleus (Sin *et al.,* (2016)). In addition, Groisman (2006) demonstrated that the degradation of CSB depends on the action of an E3-ubiquitin ligase complex that contains CSA.

**Figure 3:**
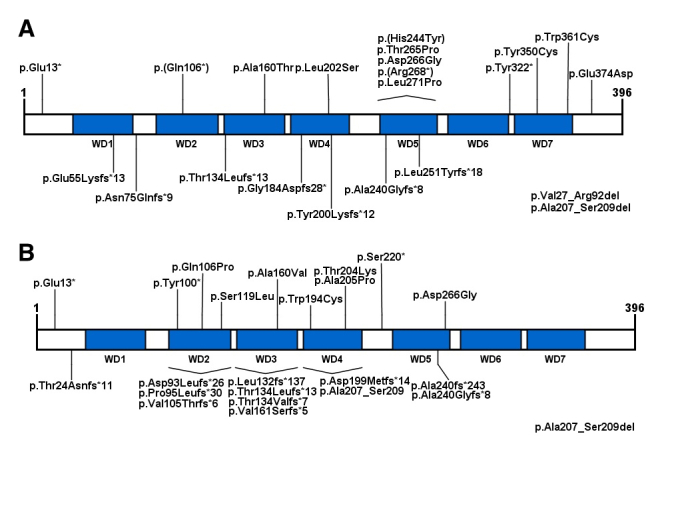
Representation of CSA protein and its seven WD-repeat domains. (A) Homozygous mutations are indicated: frameshifts and nonsense mutations are indicated above the protein, while deletions and missense mutations are indicated below the protein. (B) Represents heterozygous mutations.

CSA is a 44 kDa protein 396 amino acid residues long and is encoded by the *ERCC8* gene located in chromosomal region 5q12.1 (Henning *et al.*, 1995). It belongs to the WD-repeat family because it contains 7 WD40 domains that are repeated in its structure. These domains consist of approximately 40 amino acid residues that start with a conserved glycine and histidine sequence and terminate in tryptophan and aspartic acid (WD), a seven-bladed propeller structure with its N terminus attached to DDB1 via a helix-loop-helix motif (Fischer*et al.*, 2011). Proteins with the WD40 domain characteristically interact with other proteins and are typically known for their ability to form protein complexes (Xu and Min, 2011). Although they do not have a catalytic activity, they are involved in a variety of cellular functions, such as the regulation of transcription and chromatin conformation, apoptosis, signal transduction, and cell cycle control, among others (Xu and Min, 2011). CSA protein structure and homozygous/heterozygous pathological alterations are illustrated in Figure 3A and B. Table S2 shows all the ERCC8 mutations reported in the literature.

It is known that CSA is part of the E3 ubiquitin ligase complex, along with DDB1, RBX1, and CUL4A (Groisman *et al.*, 2003; Fischer *et al.*, 2011), responsible for the ubiquitination and degradation of TC-NER proteins when the repair is finalized (Groisman *et al.*, 2006). In addition, CSA interacts and complexes with several proteins involved in transcription, ribosomal biogenesis, and TC-NER (reviewed by Aamann *et al.*, 2014; Koch *et al.*, 2014). Nonetheless, the mechanisms via which these interactions occur, and which CSA regions are involved, are not yet completely understood.

The three-dimensional structure and conserved amino acid residues in specific regions of the protein are of great importance for the regulatory activity and the interactions. Mutations in these regions that lead to the alteration of one of these factors may inevitably impair these functions (Muftuoglu *et al.*, 2002:; Christiansen *et al.*, 2003).

To identify a possible relation between the mutations and the phenotype of patients with CS, we mapped all the mutations in the *ERCC8* and *ERCC6* genes and their impact on proteins CSA and CSB, respectively, that is already reported in the literature. In total, we found 102 mutations in *ERCC6* (50 homozygous and 52 heterozygous) and 37 mutations in *ERCC8* (23 homozygous and 14 heterozygous). These numbers indicate that 70% of all the cases of the disease are caused by CSB mutations and 30% by CSA mutations. Analyzing only homozygous mutations, we observed the lack of an obvious correlation between the type of mutation or the affected region with the severity of CS (Tables S1 and S2).

Nevertheless, as discussed by Laugel (2013), the large prevalence of type I CS clinical classification (moderate phenotype) is noted in CSA patients, with most of the mutations located in the WD domains. Regarding CSB, mutations are predominant among the domains (such as domains III and IV, for example) and are mostly nonsense mutations and frameshifts, with lower prevalence of missense mutations and deletions. Unlike CSA cases, type II CS clinical manifestation is predominant among CSB patients (Laugel, 2013; Calmels*et al.*, 2018).

The mutations that give rise to truncated CS proteins and the phenotype of the patients are also non-correlating variables. Proteins with mutations in the amino acid end sequences, such as R1087X, all have the integral ATPase domains but still lead to the manifestation of type II CS (more severe phenotype). On the other hand, the most striking example is the total absence of functional CSB resulting from the R77X mutation, as reported by Horibata *et al.* (2004), not leading to CS manifestation but instead causing UV-sensitive syndrome (UVSS) (Spivak, 2005). Another interesting observation is that the same mutation may cause different forms of CS in different patients. Other examples of one specific mutation resulting in different phenotypes exist in the literature. For example, Colella *et al.* (2000) described two siblings with the R735X mutation in CSB that do not cause CS but instead induce De Sanctis-Cacchione syndrome (a variant form of XP), whereas Mallery *et al.* (1998) described an association of the same mutation with type I of CS. In addition, Jaakkola *et al.* (2010) discovered the R1288X mutation (in CSB), which causes a severe neurological disorder known as cerebro-ocular-facial-skeletal syndrome (COFS) in several members of the same family, while the same mutation was also associated with type II CS manifestation (Laugel *et al.*, 2009). This unexpected difference in phenotypes suggests that the genetic background may be a key factor that also needs to be taken into account in studies on the development, diversification of forms, and manifestations of the disease.

## CS as a transcription syndrome

A deficiency in TC-NER and the consequent inability to recover RNA synthesis upon DNA damage in actively transcribed strands of genes could contribute to the neurological CS phenotype. Nonetheless, patients with UVSS (which can be caused by mutations in genes *CSA*, *CSB*, or *UVSSA*) lack TC-NER and are sun sensitive but do not manifest any of the neurological symptoms of CS (Itoh *et al.*, 1996; Horibata *et al.*, 2004; Spivac, 2005; Nardo *et al.*, 2009; Zhang *et al.*, 2012; Brooks, 2013).

In 1997, both the Hoeijmakers and Sancar groups discovered that CSB associates with RNA pol II, suggesting that CSB is implicated in transcription (Selby and Sancar, 1997a,b; van Gool *et al.*, 1997). In fact, CSB was shown to increase the rate of transcription elongation up to threefold in an *in vitro* assay that reconstituted the transcription system (Selby and Sancar, 1997b). Similar observations were made by Balajee *et al.* (1997). These results prompted the authors to suggest that CS may be mainly a transcription, rather than a DNA repair related syndrome.

Five years later, Bradsher *et al.* (2002) stated that CSB was found in the nucleolus in a complex containing RNA pol I, which regulates ribosomal RNA (rRNA) transcription. The authors noticed that a CSB mutant cell line showed 8- to 10-fold lower rates of rRNA synthesis than did wild-type cells, confirming a critical role of CSB not only in mRNA synthesis (by RNA pol II) but also in rRNA synthesis (by RNA pol I). Of note, the CSA protein turned out to be associated with RNA pol I in the nucleolus, and a knockdown of CSA reduced rRNA synthesis (Koch *et al.*, 2014). This malfunction in RNA pol I transcription has also been linked to endoplasmic-reticulum stress, leading to an unfolded protein response and the loss of proteostasis, which may be linked to the CS phenotype (Alupei *et al.*, 2018).

In agreement with the idea that CS is a transcription syndrome, Newman *et al.* (2006) found that CSB-null fibroblasts feature a gene dysregulation pattern similar to that induced by HDAC inhibitors. Wang *et al.* (2014) reported dysregulation of several genes (linked to neurons) in CSB-mutant and CSA-mutant fibroblasts and in *post-mortem* brain tissue of patients. The authors also noticed that *in vitro* transdifferentiation of fibroblasts into neurons and neuroblast differentiation are impaired in CSB-deficient cells. These results strongly indicate that CSB is critical for neuronal differentiation and maintenance, and that gene expression defects might underlie the neurodegenerative and the neurodevelopmental defects observed in patients.

By taking advantage of cell reprogramming, Andrade *et al.* (2012) for the first time reprogrammed CSB-mutated primary fibroblasts into induced-pluripotent stem cells. Aside from making it possible to obtain a cell type that can be used to model CS development *in vitro*, the authors noticed dysregulation of hundreds of targets (including p53 and TXNIP) in these cells (Andrade *et al.*, 2012). More recently, by combining cell reprogramming with neuron differentiation protocols, Vessoni *et al.* (2016) were, for the first time, able to obtain live neurons from patients’ skin fibroblasts. By RNA sequencing, the authors were able to find that pathological mutations in the *ERCC6* gene changed the expression of almost 5000 transcripts in neurons of CSB-deficient patients compared to unaffected controls. Pathways related to axonogenesis, the action potential of neurons, neurotransmission, as well as transcripts related to the growth hormone–IGF-1 pathway were found to be dysregulated in the CSB-deficient neurons. Collectively, these results confirm that CSB deficiency heavily impacts the transcriptional process of the cell types relevant for the disease, even in the absence of exogenous DNA-damaging agents. Such extensive transcriptional dysregulation may underlie the complex and heterogeneous CS phenotype.

Nonetheless, as mentioned before, some mutations in genes *XPB*, *XPD*, *XPF*, *XPG*, or *ERCC1*, which participate in NER (Menck and Munford, 2014), may result in a combined phenotype of CS and XP (Lehmann 1982,^ 2014^; Weeda *et al.*, 1990; Moriel-Carretero *et al.*, 2015). How can we explain the CS phenotype in all these cases? A likely answer to this question may depend on a multiprotein complex, TFIIH, which is indispensable for NER and for transcription. TFIIH consists of two functional subcomplexes (Core and CAK) that participate in initiation, promoter escape, and early elongation (Compe and Egly, 2012). The core subcomplex consists of seven proteins, including 3’ to 5’ ATP-dependent helicase XPB, and XPD, a 5’ to 3’ ATP-dependent helicase that binds to the core and CAK complexes together and facilitates optimal transcription (Tirode *et al.*, 1999; Egly and Coin, 2011). In this sense, two mutations in the *XPB* gene (associated with the XP/CS phenotype) reduce transcriptional activity in a reconstituted transcription assay *in vitro* (Coin *et al.*, 1999). Moreover, *XPD* or *XPB* mutations associated with CS were found to disrupt the interactions among CSB, TFIIH, and RNA pol I (Bradsher *et al.*, 2002). The XPG nuclease was also found to associate with and stabilize TFIIH, and mutations in the *XPG* gene related to an XP-G/CS phenotype abrogate the XPG–TFIIH interaction (Ito *et al.*, 2007; Lehmann *et al.*, 2014; Narita *et al.*, 2015). The recently described XP/CS patients with mutations in *XPF* or *ERCC1* (Kashiyama *et al.*, 2013) pose a challenge to the “CS as a transcription syndrome” point of view, because XPF/ERCC1 is implicated in NER and not in basal transcription. Although XPF is recruited to the promoter of inducible genes (to facilitate chromatin modification for transcription) in the absence of exogenous DNA damage, other NER factors are recruited as well, including XPC, in which mutations do not result in neurological abnormalities (Le May *et al.*, 2010). XPF mutations that cause the XP/CS phenotype were recently found to cause persistent recruitment of NER proteins to DNA damage sites, which may induce the stalling of RNA and DNA polymerases, thereby interfering with the transcription and replication processes (Sabatella *et al.*, 2018). Nevertheless, endogenous levels of DNA lesions that are substrates for NER have been reported to accumulate in mammalian cells and tissues to the levels that would not be consistent with the notion of defective TC-NER as a cause of the neurological symptoms of CS (Brooks, 2013). Therefore, more studies are needed to clarify the mechanism behind XPF/ERCC1 mutations and the development of the CS phenotype in these patients (Kashiyama *et al.*, 2013).

## CS and redox processes

The manifestation of CS occurs only when some NER proteins are mutated, while the complete deactivation of this pathway, via mutations in *XPA*, leads to the development of XP. Consequently, the roles of proteins CSA and CSB in addition to those known classic functions come into question. In addition, the main symptoms of patients with CS, e.g., neurological aberrations, cannot be explained only by the inefficient repair of UV damage because neurons are not exposed to this type of radiation. Due to the systemic presence of reactive species and their ability to chemically and structurally modify biomolecules, especially DNA, these compounds have been investigated as possible contributing factors of CS.

Experiments with keratinocytes derived from patients with CS suggest that these cells contain high concentrations of reactive oxygen species (ROS) with redox balance alterations under baseline conditions, characteristics that are related to the senescence phenotype of these cells (Cordisco *et al.*, 2018). The induction of redox processes by the exposure of mice and CSB^-/-^ mouse embryonic fibroblasts to ionizing radiation or paraquat revealed high sensitivity to the toxic effects of these agents, while such high sensitivity is not observed in CSA-mutant mice (de Waard *et al.*, 2003, 2004). The sensitivity to the redox processes was also observed in CSA^-/-^ keratinocytes and fibroblasts treated with potassium bromide (D’Errico *et al.*, 2007) and in CSB^-/-^ cells exposed to MMS (methyl methansulfonate) and 5-hydroxymethyl-2’deoxyuridine (Wong *et al.*, 2007).

Moreover, lipid peroxidation products such as HNE (4-hydroxynonenal) at high cellular concentrations perform direct modifications on the CSB protein, compromising its ATPase activity required for the DNA repair by TC-NER (Maddukuri *et al.*, 2009). On the other hand, Boetefuer *et al.* (2018) demonstrated that this activity is not needed for CSB–chromatin association when CS1AN-sv cells are exposed to menadione. In that condition, these associations are loci-specific and are stimulated by the PARP1 protein, thereby indicating possible participation of CSB in the transcriptional regulation in response to oxidative stress (Boetefuer *et al.*, 2018a). These findings are in agreement with previous results that point to the involvement of a CSB function in this process, thereby showing that CSB localization and interaction with transcriptional repressor CTCF in promoter regions are greater in cells under oxidative stress (Lake *et al.*, 2016; Boetefuer *et al.*, 2018b).

Although DNA oxidation products are typically repaired by the BER pathway, it has been demonstrated that BER and NER proteins not only show crosstalk, but also that some oxidatively generated lesions are substrates for the NER pathway (D’Errico *et al.*, 2007; Berra *et al.*, 2013). For example, 8-oxoGua, thymine glycol, malondialdehyde, and etheno adducts induce distorting modifications in the double helix and have the potential to block transcription (reviewed by Tornaletti, 2005; Chaim *et al.*, 2017), with cyclopurines being DNA oxidation products that are repaired only by NER (Brooks *et al.*, 2000). Numerous experiments have confirmed the importance of the CSA and CSB proteins for the repair of oxidized bases, by demonstrating that in the absence of CSA or CSB there is accumulation of 8-oxoGua in DNA (Dianov *et al.*, 1999; Tuo *et al.*, 2001; D’Errico *et al.*, 2007; Aamann *et al.*, 2014a; Cordisco *et al.*, 2018). 8-OH-Ade, 5-hydroxycytosine, and cyclopurines are also lesions that are inefficiently repaired in cells harboring mutations in CS genes (Tuo *et al.*, 2002b, 2003; D’Errico *et al.*, 2013). Because of the inefficient repair in the cells of important tissues such as the brain, the accumulation of these lesions in DNA may be one of the factors that cause and aggravate the neurological symptoms of the disease, as seen in patients with neurodegenerative diseases, such as Alzheimer’s, Parkinson’s, Huntington’s disease, and amyotrophic lateral sclerosis (Ayala-Peña, 2013; Coppedè *et al.*, 2016; Abolhassani *et al.*, 2017).

The mutational impact of oxidatively induced DNA damage in CS was investigated by Lodato *et al.* (2018). By means of single-cell whole-genome sequencing followed by genome-wide somatic single-nucleotide variant identification, they detected an increased number of mutations in the neurons of patients with CS as compared to the control. In addition, C > A variants, a signature for mutations induced by oxidatively induced DNA damage, are found in a higher frequency among patients with CS (Lodato *et al.*, 2018).

It is noteworthy that in response to irradiation or hydrogen peroxide, CSA is translocated to the nuclear matrix by a CSB-dependent mechanism (Kamiuchi *et al.*, 2002), but as discussed above, these two proteins perform different functions in chromatin remodeling and in recruitment of the factors associated with the repair and blocking of RNA pol II *in vivo* (Fousteri *et al.*, 2006). This cooperation between CSA and CSB was not observed in response to treatment with alkylating agents and is independent of XPA and XPC, thus suggesting that TC-NER plays a key part in this cellular response mechanism (Kamiuchi *et al.*, 2002).

Brain biopsies of CS and XPA patients yielded distinct results in response to the accumulation of oxidized bases in DNA and SOD expression, with only XPA-mutant patients showing upregulation of 8-oxoGua in the nucleus and alteration in SOD expression (Hayashi *et al.*, 2005).

In addition to the participation of these proteins in the repair of oxidation-induced lesions through the NER pathway, there is also a contribution to the removal of DNA damage via direct and indirect activity in the BER pathway (Khobta and Epe, 2013). This contribution is mediated by the direct modulation through interactions of CSB with BER protein glycosylases, APE1, NEIL1 and NEIL2, and association with the OGG1 complex, which stimulates the incision activity of these proteins and drives the repair (Wong *et al.*, 2007; Muftuoglu *et al.*, 2009; Aamann *et al.*, 2014b). Csb^m/m^/Ogg1^-/-^ mice show high concentrations of 8-oxoGua in comparison with Ogg1^-/-^ animals, thereby confirming the importance of the cooperation between these two proteins in the removal of these lesions (Osterod *et al.*, 2002; Trapp *et al.*, 2007). Pastoriza-Gallego *et al.* (2007) also demonstrated that the 8-oxoGua lesions exert different effects on gene expression depending upon the promoter and sequence context, and that both proteins, Csb and Ogg1, are required for full repair.

Recent evidence also indicates that CSB recruits the XRCC1 protein to single-strand break regions after oxidatively induced DNA damage for subsequent processing by BER (Menoni *et al.*, 2018). In the indirect modulation of the BER pathway, CSB acts as an important factor in the expression of *hOGG1*, inducing its transcription. It has also been observed that in CSB-deficient cells, the amounts of *OGG1* mRNA and protein are low (Dianov *et al.*, 1999; Tuo *et al.*, 2002a; Aamann *et al.*, 2014b).

Some studies point to a possible implication of CSB in the repair of oxidized bases in genomic and mitochondrial DNA, to CSB migration to mitochondria, and modulation of p53 activity in response to a redox process (Stevnsner *et al.*, 2008; Frontini and Proietti-De-Santis, 2009; Aamann *et al.*, 2010).

The repair of oxidized bases also has a fundamental role for the mitochondria. These organelles produce ATP through the electron transport chain, a process that not only provides cells with energy, but also produces ROS as a by-product of respiration (reviewed by Kowaltowski *et al.*, 2009). For this reason, mitochondria are also considered the primary source of ROS in cells, having a much more oxidizing environment within the mitochondrial matrix as compared with the cellular cytosol (Hu *et al.*, 2008).

Because of proximity to the initial site of formation and constant exposure to ROS, mitochondrial DNA (mtDNA) is the main target of these molecules, and just as nuclear DNA, it is susceptible to oxidation in the DNA bases and undergoes other modifications in its structure (reviewed by Muftuoglu *et al.*, 2014). Besides, the accumulation of lesions in mtDNA causes instability and compromises its functioning, which can lead to mutations and affect genes that are important for mitochondrial metabolism. In addition, various aging-related diseases, such as cardiovascular diseases, Parkinson’s disease, Alzheimer’s disease, and cancer are associated with significant amounts of lesions in mitochondria, thus being considered one of the causes of the pathologies related to mitochondrial dysfunction (Mecocci *et al.*, 1994; Ide *et al.*, 2001; Stichel *et al.*, 2007; Wallace, 2012).

Mitochondria have some specific repair pathways, such as BER and mismatch repair (MMR). As in the repair of nuclear DNA, the CSA and CSB proteins are present and participate in this process, even though there is no mitochondrial NER (Kamenisch *et al.*, 2010). It has been demonstrated that CSA and CSB, after induction of redox processes by the exposure to H2O2, UV, or menadione, are directed toward mitochondria and interact with OGG1 (Aamann *et al.*, 2010; Kamenisch *et al.*, 2010; reviewed by Prates Mori and de Souza-Pinto, 2018). In mitochondria, CSB may act as a modulator of the BER pathway, in association with the inner mitochondrial membrane for mtDNA lesion removal (Stuart *et al.*, 2005; Aamann *et al.*, 2010). In fact, CSB^m/m^ mice accumulate 2.5-fold more 8-oxoGua in mtDNA than wild-type animals (Osenbroch *et al.*, 2009). The mutation in CSB^m/m^ mice is the same as that in the *ERCC6* gene of the CS1AN patient, K337 → stop, which generates a truncated nonfunctional protein (Troelstra *et al.*, 1992; van der Horst *et al.*, 1997).

In addition to the influence on DNA repair, CSB is involved in gene expression by engaging in the elongation by RNA polymerase II (Selby and Sancar, 1997a). In *in vitro* transcription, through interactions with proteins of the mitochondrial nucleoid complex, CSB promotes POLRMT transcriptional elongation and allows it to access mtDNA by TFAM removal from the region to be transcribed (Berquist *et al.*, 2012).

Regarding CS mitochondrial metabolism, Scheibye-Knudsen *et al.* (2012) reported a significant increase of this metabolism in both CSB^m/m^ mice and CSB-deficient cells, and this phenomenon is also manifested in CSA- or CSB- deficient fibroblasts and CSA^-/-^/XPA^-/-^ mice (Pascucci *et al.*, 2012; Brace *et al.*, 2016). This increase in energy metabolism is accompanied by a large production of ROS through electron leakage (Pascucci *et al.*, 2012; Cleaver *et al.*, 2014), and is the main source of DNA damage induction (Scheibye-Knudsen *et al.*, 2012) and of changes in the membrane potential, excessive fragmentation, and mitochondrial fission (Pascucci *et al.*, 2012, 2016).

Under normal cell conditions, organelles and defective proteins are eliminated by autophagy. On the other hand, dysfunctional or damaged mitochondria are subjected to a special form of autophagy, called mitophagy (Fivenson *et al.*, 2017). This mechanism contributes to cell homeostasis, and its malfunction is present in several aging-related diseases (reviewed by Redmann *et al.*, 2014). Human and murine CSB-mutant fibroblasts feature a dysfunction in this pathway owing to defects in the recruitment of P62, an essential player in mitophagy (Scheibye-Knudsen *et al.*, 2012).

Thus, dysfunctional mitochondria, with large amounts of mtDNA lesions, can produce more ROS via their metabolism. Consequently, ROS accumulate in the cells, promoting apoptosis (Pinto and Moraes, 2015; van Houten *et al.*, 2016), which might be associated with reduced amounts of subcutaneous fat in CSA^-/-^ and CSB^m/m^ mice (Kamenisch *et al.*, 2010). Treatment with pharmacological activators of autophagy, such as rapamycin (Scheibye-Knudsen *et al.*, 2012) and an HDAC inhibitor (Majora *et al.*, 2018), attenuates the accumulation of damaged mitochondria in CSB-deficient cells and the loss of subcutaneous fat in CSB^m/m^ mice.

## Models for CS study

To better understand the mechanisms of CS progression, several animal models have been developed for research on the disease, e.g., mice, *Caenorhabditis elegans*, and zebrafish. In general, such models carry knockout mutations or mutations identical to those found in humans, enabling a more accurate analysis in different cells and tissues of an organism.

The first models generated to study CS were created on the basis of mice with the C57BL/6 background - CSB^m/m^ mice (van der Horst *et al.*, 1997), and CSA^-/-^ mice (van der Horst *et al.*, 2002). In CSA^-/-^ cells, the interruption of the gene sequence in exon 2 of the *Ercc8* gene results in protein absence (van der Horst *et al.*, 2002). When exposed to UV, the fibroblasts of these animals manifest higher UV sensitivity, and they have an inability to resume RNA synthesis and defective TC-NER, these being the classic characteristic of CS (Lehmann, 1982; van der Horst *et al.*, 1997, 2002).

The CSB^m/m^ and CSA^-/-^ models have similar phenotypic characteristics, although neither has the severe neurodegenerative phenotype and lifespan reduction seen in patients with CS. Despite differences between them, these mouse models have a few characteristics that resemble their human counterpart, such as the tendency toward reduced body weight via a decrease in the amounts of subcutaneous and visceral fat (Gorgels *et al.*, 2007; Scheibye-Knudsen *et al.*, 2012), mild neurological dysfunction with changes in myelin (Jaarsma *et al.*, 2011), activation of glial cells in white-matter regions (Jaarsma *et al.*, 2011), progressive deafness (Nagtegaal *et al.*, 2015), photophobia and sensitivity to UV exposure (van der Horst *et al.*, 1997*,* 2002). In contrast to what is manifested in humans, the mouse models are prone to skin and eye cancers when exposed to UV, a feature that can be explained by the inefficiency of murine GG-NER in dealing with UV-induced cyclobutane pyrimidine dimer-like lesions, which are preferentially repaired by TC-NER (van der Horst *et al.*, 1997, 2002; de Boer and Hoeijmakers, 1999).

CSB^m/m^ cells (from several tissues, mainly the brain and kidneys) also contain high levels of formamidopyrimidines and 8-oxoGua in DNA when compared to wild-type mice (Muftuoglu *et al.*, 2009). In addition to nuclear DNA damage, mtDNA damage and mutational load in the mtDNA of CSA^-/-^ and CSB^m/m^ mice increased during aging and is related to subcutaneous fat loss, one of the main characteristics of CS, probably mediated by the apoptosis caused by mitochondrial dysfunction (Kamenisch *et al.*, 2010) and by cell senescence, a feature commonly present in progeroid models (Carrero *et al.*, 2016).

The combined deletion of other NER proteins such as XPA in these CS mice leads to a more severe phenotype. These animals manifest severe neurological symptoms, ataxia, compromised growth, low weight, a lack of motor coordination, kyphosis, and abnormal behavior and development with premature death within 20 days (Murai *et al.*, 2001; van der Pluijm *et al.*, 2007). Inactivation of GG-NER by an XPC knockout in CSB^m/m^ mice leads to a similar phenotype, with dysmyelination, slow development, and low body weight: characteristics that resemble those of patients with CS (Laposa *et al.*, 2007; van der Pluijm *et al.*, 2007; Revet *et al.*, 2012). CSA^-/-^/XPA^-/-^ mice also show evidence of neurological problems, life expectancy reduced to ~1 month, an increase in cellular oxidative phosphorylation, and lipodystrophy (Brace *et al.*, 2013, 2016).

The mechanism that underlies this aggravation of the CS phenotype by the double inactivation of CS and XP genes in mice has yet to be uncovered. Since CS and XP proteins act in the same DNA repair pathway, it is assumed that this severe CS phenotype in mice is due to the disruption of both NER-related and other functions of the CS proteins, such as transcription regulation (Brooks, 2013) and/or interactions with other DNA repair pathways (Murai *et al.*, 2001). Inactivation of NER by an *XPC* or *XPA* gene knockout can result in the accumulation of transcription-blocking DNA lesions, and the loss of either CS protein may further interfere with transcription and further increase the DNA damage load, which can in turn have detrimental effects, such as cell death or early cell senescence, both of which have been observed in progeroid mice (Weeda *et al.*, 1997; van der Pluijm *et al.*, 2007; Brooks, 2013).

Combined inactivation of TC-NER and NER in neurons has also been reported to generate an age-related progressive neurodegenerative phenotype. CSB^m/m^ mice featuring a neuron-specific conditional XPA knockout have a shorter lifespan, behavioral abnormalities, and brain atrophy. These characteristics are possibly related to a synergistic effect of the TC-NER and NER pathways or to other functions of the inactivated proteins, such as chromatin remodeling or other functions in transcription (Newman *et al.*, 2006; Jaarsma *et al.*, 2011; Wang *et al.*, 2018). Cell type–specific conditional knockouts may also help to better understand the participation of other processes in the progression of the disease, along with their molecular mechanisms, e.g., the role of oligodendrocytes (Howng *et al.*, 2010) or other glial cells (Raj *et al.*, 2014) in CS dysmyelination and in the neurodegenerative phenotype.

Mutations in XPG or ERCC1 can yield mice with the characteristics similar to those of CS and other diseases on the CS spectrum, such as XP/CS and COFS (Jaarsma *et al.*, 2013). These NER nuclease–deficient models have several neurodegenerative features, altered metabolism, and a reduced lifespan (Weeda *et al.*, 1997; Barnhoorn *et al.*, 2014). Notably, despite the lipodystrophy observed in all these animal models, dietary restriction and methionine restriction (which are metabolic interventions known to increase the lifespan of several species) reversed some of the neurodegeneration indicators of the CSA^-/-^/XPA^-/-^ and ERCC1^Δ/-^ models, with dietary restriction nearly doubling the lifespan of ERCC1^Δ/-^ mice. This finding indicates that metabolic interventions are a possible therapy for NER-related progeroid diseases (Brace *et al.*, 2016; Vermeij *et al.*, 2016).

The current murine models for CS research do not fully reproduce the phenotype seen in humans because these animals manifest milder symptoms of the disease when only a single TC-NER protein is mutated. One of the possible explanations is the adaptation of NER to deal with DNA damage that would be repaired initially by CSA and CSB. This explains the severe symptoms when both TC-NER and NER are inactivated (van der Pluijm *et al.*, 2007). In this case, it is important to consider the evolutionary distance that separates mice from humans. This distance entails differences in gene expression and metabolic and physiological profiles, among others (Seok *et al.*, 2013; Lin *et al.*, 2014). Such differences contribute to the following phenomenon: the phenotype resulting from the same mutation and the activity of the repair pathway are not identical between the two species. Table 1 summarizes the mouse models carrying mutations in CS genes and other single-NER-mutation models that recapitulate CS-like phenotypes.

**Table 1:** Reported neurological and metabolic characteristics of Cockayne Syndrome and related mouse models.

Mouse model	Genetic background	Neurological abnormalities	Metabolic characteristics	Lifespan	Other reported features	References
Csb^m/m^	C57BL/6J	Mild, progressive neurodegeneration with no behavioral phenotype, mild astro and microgliosis, hearing loss associated with loss of neurosensorial cells, late retinal degeneration	Increased metabolism and mitochondrial numbers, Progressive lipodistrophy	> 2 years	Increased skin cancer predisposition after UV exposure	Van der Horst *et al*., 1997; Jaarsma *et al*., 2011
Csa^-/-^	C57BL/6J	Mild, progressive neurodegeneration with no behavioral phenotype, mild astro and microgliosis, hearing loss associated with loss of neurosensorial cells	Increased metabolism	> 2 years	Increased skin cancer predisposition after UV exposure	van der Horst *et al*., 2002; Gorgels *et al*, 2007; Jaarsma *et al*., 2011; Brace *et al*., 2016
Csb^m/m^/Xpa^-/-^	C57BL/6J; CBA/C57BL6/CD-1/129Ola hybrid	Profound early postnatal ataxia, abnormal cerebellar development and degeneration, loss of purkinjecells, progressive neurological dysfunction, motor coordination problems, early retinal degeneration	Severe post-natal growth deficiency, Severe lipodistrophy, decreased oxidative metabolism	3 weeks	Suppression of the GH/IGF1 somatotroph axis increased antioxidant responses, hypoglycemia	Murai *et al*., 2001, van der Pluijm *et al*., 2007; Jaarsma *et al*., 2011
Csb^-/-^ / Xpa^c/-^ CamKIIα-Cre	C57BL/6J	Progressive neurological dysfunction, Purkinje cell death, neurodegeneration, motor coordination problems starting at 6 months	Weight reduction in adult animals	90 weeks	-	Jaarsma *et al*., 2011
Csb^m/m^/Xpc^-/-^	C57BL/6J, 129Ola/C57BL/6J hybrid	Depletion of purkinje cells, neurodegeneration in cerebellum, motor coordination problems, dysmielination	Severe post-natal growth deficiency, Severe lipodistrophy	3 weeks	-	van der Pluijm *et al*., 2007; Laposa *et al*., 2007; Revet *et al*., 2012
Csb^m/m^/Ogg1^-/-^	C57BL/6J	No neurological phenotype has been observed	Not described	> 2 years	Accumulation of oxidative stress related DNA damage and mutations in various tissues	Osterod *et al*., 2002; Pastoriza-Gallego *et al*., ?2007; Fusser *et al*., 2011
Csa^-/-^/Xpa^-/-^	C57BL/6J	Early, progressive neurological dysfunction, dysmyelination, abnormal cerebellar development and degeneration, motor coordination problems, severe astro and microgliosis	Post-natal growth deficiency, increased metabolism, severe lipodistrophy, senescence of fat tissue, no fat tissue inflammation	20 weeks	-	Brace *et al*., 2013; Brace *et al*., 2016
Xpg^-/-^	C57BL/6J	Purkinje cell degeneration, cerebellum neurodegeneration	Severe post-natal growth deficiency, Severe lipodistrophy	3 weeks	Abnormalities of the gastro-intestinal tract, early fibroblast senescence	Harada *et al*., 1999; Sun *et al*., 2001; Barnhoorn *et al.*, 2014
Xpg^-/-^	C57BL6/FVB F1 hybrid	Purkinje cell death, neurodegeneration, motor coordination problems axonal spheroids, severe astrogliosis	Post-natal growth deficiency, loss of subcutaneous fat	18 weeks	Skeletal abnormalities, mild anisokaryosis, increased mean nuclear size of liver cells, supression of GH/IGF1 axis	Barnhoorn *et al*, 2014; Vermeij, 2016
Ercc1^-/-^	Hybrid C57Bl/6/129	Early, progressive neurological dysfunction, neurodegeneration and motor coordination problems	Severe post-natal growth deficiency, decreased oxidative metabolism, severe lipodistrophy, increase in fat tissue inflammation	3 weeks	Liver nuclear abnormalities, p53 upregulation in liver and kidney, early fibroblast senescence	Mcwhir *et al*., 1993, Weeda *et al*., 1997
Ercc1^Δ/-^	C57Bl6J/FVB hybrid	Early, progressive neurological dysfunction, myelin abnormalities, abnormal cerebellar development and degeneration, Motor coordination problems, severe astro and microgliosis	Post-natal growth deficiency, decreased oxidative metabolism, severe lipodistrophy, increase in fat tissue inflammation	22-25 weeks	Early fibroblast and vascular tissue senescence, reduced bone density	Weeda *et al*., 1997; Nevedomskaya *et al*., 2010; de Waard *et al*., 2010; Durik *et al*., 2012; Vermeij *et al.*, 2016;

Different animal models of lower complexity have also been developed to study the role of CSA and CSB proteins, and are an interesting alternative for the research into protein functions. Among these models are *Caenorhabditis elegans* and zebrafish.

*C. elegans* has advantages, such as the ease of laboratory maintenance, of genetic manipulation, of tissue differentiation, rapid reproduction with several offspring, a generally fixed and genetically determined number of cells, and a short life cycle, allowing for the study of its development within short periods. It has been demonstrated that the NER pathway is well conserved in C. elegans, resembling the repair mechanisms of mammals (Meyer *et al.*, 2007; reviewed by Lans and Vermeulen, 2011), and because they do not repair DNA by photoreactivation (Hartman *et al.*, 1989). Rather, the lesions caused by UV are repaired exclusively by NER. CSA and CSB analogs, csa-1 (Babu *et al.*, 2014) and csb-1 (CeCSB) (Lee *et al.*, 2002), respectively, were found to be a part of this pathway. Animals mutated in csa-1 are hypersensitive to UV-B light exposure (Babu *et al.*, 2014), and csb-1–deficient animal germ cells show apoptosis induction and morphological abnormalities after exposure to this agent (Lee *et al.*, 2002). Moreover, a knockout of either csa-1 or csb-1 in *C. elegans* resulted in increased oxygen consumption and in changes in the transcription of genes related to mitochondrial ATP production, ubiquitin pathways, and transcriptional regulation (Scheibye-Knudsen *et al.*, 2016).

In general, *C. elegans* is a good model for the investigation of the DNA damage response to UV via the NER pathway, because this species makes it possible to reproduce and examine the effects of irradiation or other DNA-damaging agents on an entire organism and throughout its developmental stages within a short period of time.

The zebrafish is a vertebrate model that is widely used in research on the effects of exposure to genotoxic agents, carcinogenesis processes, and mainly embryonic development (Spitsbergen and Kent, 2003; Titus *et al.*, 2009). This animal has orthologous DNA repair genes in the pathways present in higher eukaryotes, e.g., BER, NER, MMR, Non-homologous end joining and Homologous recombination (Pei and Strauss, 2013). In NER, 44 genes are responsible for the functioning mechanisms of damage removal. Despite this observation, there are still few zebrafish studies where these characteristics are exploited from the perspective of DNA repair alone.

Zebrafish with the CSB depleted by morpholino oligonucleotides at the larval stage show an increased frequency of morphological abnormalities, which may recapitulate some of the congenital and developmental manifestations seen in patients with CS. Ionizing radiation can further increase morphological aberrations in CSB-depleted zebrafish, thereby pointing to an important role of CSB in the defense against oxidative DNA damage (Wei *et al.*, 2015).

Due to the complexity of CS and the unique characteristics of patients with CS, which cannot be fully recapitulated in any animal model, a combination of human cell models with various animal models offers complimentary approaches to elucidate the various characteristics of this syndrome. In this sense, the use of somatic cell reprogramming coupled with genome editing allows investigators to obtain relevant and functional cell types (such as neurons) carrying patient-specific mutations. Moreover, this method offers an opportunity to investigate how the genetic background of different patients interacts with the one given pathological mutation. Such an approach may clarify why certain patients that share the same mutation in a CS gene have different phenotypes (Colella *et al.*, 2000).

## Conclusion

Almost 60 years of accumulated research in the NER field, especially regarding progeroid CS, provides extensive knowledge about the structure of proteins CSA and CSB and their participation in TC-NER and other mechanisms, such as transcription, repair, and mitochondrial functioning. For the advances made so far, human cellular models and animal models have been invaluable. They have revealed that the CS phenotype is likely a combination of altered gene transcription, metabolic adjustment, redox imbalance, and DNA repair defects, although the relative importance of each of these mechanisms for the disease is still largely unknown. Moreover, the full spectrum of the syndrome is not completely mirrored in animals, and this situation undoubtedly hinders further research. This difficulty is also associated with the impossibility of correlation between the mutations in *ERCC6*/*ERCC8* genes and the phenotype of the patients, suggesting that the genetic background can heavily influence the manifestation of the symptoms.

Recently, the ability to generate pluripotent stem cells from patients with CS enabled for the first time the recapitulation of the full genetic signature of a patient in a cell type relevant for the disease, e.g., neurons. This observation expands and complements the previous CS models because it enables the investigation of metabolism, of the DNA damage response, and of gene expression in a patient’s cells. These data were previously impossible or very difficult to obtain. Still, one needs to keep in mind that CS is a systemic disease, and for this reason, investigating how dysfunction in one tissue/organ impacts others is critical. All these difficulties reveal how complex and diverse CS is and imply that a multimodel approach will therefore help to better recapitulate certain characteristics of CS. Moreover, although not entirely equivalent to normal aging, CS has clinical and cellular similarities to some aging-related diseases. Thus, improvements of (and new approaches to) CS models may have a broad impact on the study of these diseases as well.

## Supplementary material

The following online material is available for this article:

Table S1 - Homozygous and heterozygous ERCC6 (CSB) mutations and their effects on patients phenotype.

Table S2 - Homozygous and heterozygous ERCC8 (CSA) mutations and their effects on patients phenotype.
