# Supplementary material for: Cockayne Syndrome: The many challenges and approaches to understand a multifaceted disease
Source: Genet Mol Biol. 2020 May 20;43(1 Suppl 1):e20190085. doi: 10.1590/1678-4685-GMB-2019-0085 (PMC7250278; doi:10.1590/1678-4685-GMB-2019-0085)
Supplement: Table S1 [file 1415-4757-GMB-43-1-s1-e20190085-suppl1.pdf]

Supplementary Material to “Cockayne Syndrome: The many challenges and approaches to understand a multifaceted disease”

Table S1 - Homozygous and heterozygous *ERCC6 (CSB*) mutations and their effects on patients phenotype.

***CSB* - Homozygous**			
**Mutation**	**Patient code**	**CS type**	**Reference**
p.Arg735*	CS17LO<br>CS128ST	CS I	(Calmels *et al.*, 2018)
p.Arg652*	CS10X	?	(Calmels *et al.*, 2018)
p.(Gln854*)	CS144ST	CS II	(Calmels *et al.*, 2018)
p.(Asp1355Valfs*32)	CS071ST<br>CS222ST	CS I	(Calmels *et al.*, 2018)
	CS204ST<br>CS221ST	CS III	(Calmels *et al.*, 2018)
p.Leu72Cysfs*12	CS18BR	CS I	(Calmels *et al.*, 2018)
p.(Glu214*)	CS010ST	CS I	(Calmels *et al.*, 2018)
p.Ser357*	CS13PV	CS I	(Calmels *et al.*, 2018)
p.(Thr377Glnfs*28)	CS201ST	CS II	(Calmels *et al.*, 2018)
p.Lys478Thrfs*9	CS19PV	CS II	(Calmels *et al.*, 2018)
p.Trp517*	CS4TAN<br>CS8TAN	CS II	(Calmels *et al.*, 2018)
p.(Glu564*)	CS107ST	CS II	(Calmels *et al.*, 2018)
p.(*Asp646Asn*)	CS253ST	CS II	(Calmels *et al.*, 2018)
p.Arg652*	CS13MA	CS II	(Calmels *et al.*, 2018)
p.(Thr659Cysfs*24)	CS232ST	CS II	(Calmels *et al.*, 2018)
p.Phe665Tyrfs*18	CS1PL	CS III	(Calmels *et al.*, 2018)

p.Gly715*	CS23PV	CS I	(Calmels *et al.*, 2018)
p.Phe665_Gln723del	CS11MA	CS II	(Calmels *et al.*, 2018)
p.Asn760Lysfs*2	CS8PV	CS II	(Calmels *et al.*, 2018)
p.Met867Thrfs*14	CS31PV	CS II	(Calmels *et al.*, 2018)
p.Leu875Pro	CS20PV	CS II	(Calmels *et al.*, 2018)
p.Ala944Thrfs*10	CS11LO	CS II	(Calmels *et al.*, 2018)
p.Gln976Trpfs*70	CS23BR	CS II	(Calmels *et al.*, 2018)
p.(Thr1018Asnfs*32)	CS210ST	CS II	(Calmels *et al.*, 2018)
p.[Gln156*; Ser142Asnfs*4]	CS14LO	CS I	(Calmels *et al.*, 2018)
p.[Arg176*;Ser142Asnfs*4]	CS27PV	CS I	(Calmels *et al.*, 2018)
p.(Ser429Lysfs*7)	CS10LO	CS I	(Calmels *et al.*, 2018)
p.Val161Serfs*5	CS1BR	?	(Calmels *et al.*, 2018)
p.(Tyr1179Leufs*22)	CS241ST	?	(Calmels *et al.*, 2018)
p.[Gln156*; Ser142Asnfs*4]	CS3SH	?	(Calmels *et al.*, 2018)
p.Arg670Trp	?	CS II	(Chebly *et al.*, 2018)
p.Arg453*	CS1PV<br>CS3PV	CS II	(Colella *et al.*, 1999)
p.Arg735*	GM10905<br>GM10903	DSC	(Colella *et al.*, 2000)
p.Lys345Asnfs*24	CSB1NH	CS II	(Falik-Zaccai *et al.*, 2008)
p.Arg77Ilefs*6	KPSX6	CS III	(Hashimoto *et al.*, 2008)
p.Arg77*	UVS1KO	UVSS	(Horibata *et al.*, 2004)
p.Arg1288*	XVI-1<br>XVI-2<br>XIII-6<br>XIII-7<br>XIV-2<br>XIV-6	COFS	(Jaakkola *et al.*, 2010)

p.Lys971Tryfs*14	?	CS II	(Kou *et al.*, 2018)
p.Arg683*	CS789VI	COFS	(Laugel *et al.*, 2008)
p.Val763_Gln794del	CS514VI<br>CS385VI	CS II	(Laugel *et al.*, 2009)
p.Arg652*	08TR4	CS II	(Laugel *et al.*, 2009)
p.Arg1288*	CS817VI	CII	(Laugel *et al.*, 2009)
p.Arg1087*	CS6BI<br>CS8BI	CS II	(Laugel *et al.*, 2009)
p.Gln854*	CS9MA	CS II	(Laugel *et al.*, 2009)
p.Glu218Glyfs*4	AENT4	CS I	(Laugel *et al.*, 2009)
p.Asp1355Valfs*32	CS1SACT	CS I	(Laugel *et al.*, 2009)
p.Glu182Asnfs*4	CS799VI	CS III	(Laugel *et al.*, 2009)
p.Gly715*	08STR1	CS II	(Laugel *et al.*, 2009)
p.[Arg176*;Gly399Asp; Ser1321Cys]	?	CS III	(Luo *et al.*, 2014)
p.Trp517*	CS2TAN	CS I	(Mallery *et al.*, 1998)
p.Arg735*	CS1TAN	CS I	(Mallery *et al.*, 1998)
p.Trp851Arg	CS3TAN	CS I	(Mallery *et al.*, 1998)
p.Val957Gly	CS1IAF	CS I	(Mallery *et al.*, 1998)
p.Lys1239Glufs*2	?	COFS	(Meira *et al.*, 2000)
p.Arg637Serfs*34	?	CS III	(Swartz *et al.*, 2015)
p.Pro934Thr	?	CS I	(Wilson *et al.*, 2016)
p.Trp936Cys	?	CS II	(Wilson *et al.*, 2016)
p.Gln463*	?	CS I	(Zhang *et al.*, 2011)
***CSB* - Heterozygous**			
**Mutation**	**Patient code**	**CS type**	**Reference**

p.*Ser687Leu*<br>p.Arg1288*	CS278ST	CS II	(Calmels *et al.*, 2016)
p.[Gln156;Ser142Asnfs*4]<br>p.Leu471Glnfs*16	CS1SH	CS I	(Calmels *et al.*, 2018)
p.[Arg176*;Ser142Asnfs*4]<br>p.Arg652*	CS1SO	CS I	(Calmels *et al.*, 2018)
p.[Arg176*;Ser142Asnfs*4]<br>*p.Trp589del*	CS1GO	CS I	(Calmels *et al.*, 2018)
p.Ser142Asnfs*4<br>p.Arg735*	CS26PV	CS III	(Calmels *et al.*, 2018)
p.Arg453*<br>p.Val763Ilefs*7	CS1WR	CS II	(Calmels *et al.*, 2018)
p.Arg467_Arg562del<br>p.Arg1318Glyfs*12	CS058ST	CS I	(Calmels *et al.*, 2018)
p.Tyr510_Arg562del<br>p.Glu608_Gln723del	CS18PV	CS II	(Calmels *et al.*, 2018)
p.Phe563Argfs*3<br>p.[Gln943*; Gln943Profs*8; Asp904_Gln943del]	CS17PV	CS I/II	(Calmels *et al.*, 2018)
p.0<br>p.Phe665_Gln723del	CS10MA<br>CS1LI	CS II	(Calmels *et al.*, 2018)
	CS2BL	?	(Calmels *et al.*, 2018)
p.*Arg670Trp*<br>p.Val763_Gln794del	CS18LO	CS II	(Calmels *et al.*, 2018)
p.Arg683*<br>p.Phe665_Gln723del	CS12MA	CS II	(Calmels *et al.*, 2018)
p.*Ser687Leu*<br>p.Arg735*	CS1GGO	CS II	(Calmels *et al.*, 2018)
p.Leu700Valfs*60<br>p.Arg735*	CS22PV<br>CS28PV	CS I	(Calmels *et al.*, 2018)
p.Phe665_Gln723del<br>p.Arg1221*	CS8MA	CS II	(Calmels *et al.*, 2018)
p.Phe665_Gln723del<br>p.Arg1087*	CS19LO	CS II	(Calmels *et al.*, 2018)

p.Arg735*<br>p.Lys1203fs	CS2GR	CS I	(Calmels *et al.*, 2018)
p.*Trp851Arg*<br>p.Arg1318Glyfs*12	CS21PV	CS I	(Calmels *et al.*, 2018)
p.0<br>p.Arg612*	CS195ST	CS II	(Calmels *et al.*, 2018)
p.[Gln156*;Ser142Asnfs*4]<br>p.Arg947*	CS5MA	?	(Calmels *et al.*, 2018)
p.[Gln156*;Ser142Asnfs*4]<br>p.Arg735*	CS2LE	III?	(Calmels *et al.*, 2018)
p.Phe563Argfs*3<br>p.Arg735*	CS25PV	?	(Calmels *et al.*, 2018)
p.Arg683*<br>p.Arg1288*	CS32LO	?	(Calmels *et al.*, 2018)
p.Phe665_Gln723del<br>p.*Gln953Lys*	CS3BL	?	(Calmels *et al.*, 2018)
p.Arg735*<br>p.0	CS14PV	CS I	(Calmels *et al.*, 2018)
p.Ala944Thrfs*10<br>p.Tyr1179Leufs*22	CS1BEL	?	(Calmels *et al.*, 2018)
p.Arg612*<br>p.Arg975Trp	?	CS II	(He *et al.*, 2017)
p.Arg857*<br>p.Arg1087*	CS816VI	COFS	(Laugel *et al.*, 2008)
p.Leu871Pro<br>p.Lys1172*	CS881VI	COFS	(Laugel *et al.*, 2009)
p.Leu987Pro<br>p.Met752_Gln762del	CS797VI	COFS	(Laugel *et al.*, 2009)
p.Val724_Gln762del<br>p.Trp686Cys	CS683VI	CS II	(Laugel *et al.*, 2009)
p.Gln723*<br>p.Leu860del	CS3LE	CS II	(Laugel *et al.*, 2009)
p.Gly715*<br>p.Arg857*	08STR1	CS II	(Laugel *et al.*, 2009)

p.Val724_Gln762del<br>p.Ala944Thrfs*10	CS1GL	CS II	(Laugel *et al.*, 2009)
p.Met867Thrfs*14<br>p.Lys1198Argfs*4	CS117VI	CS II	(Laugel *et al.*, 2009)
p.Gln956Argfs*7<br>p.Ser687Leu	CS360VI	CS II	(Laugel *et al.*, 2009)
p.Val417Serfs*7<br>p.0	CS179VI	CS II	(Laugel *et al.*, 2009)
p.Gln156*<br>p.His1263Glnfs*67	CS21BR	CS I	(Laugel *et al.*, 2009)
p.Trp236*<br>p.Pro500Glnfs*43	CS493VI	CS I	(Laugel *et al.*, 2009)
p.Arg68Profs*13<br>p.Asn680Asp	CS784VI	CS I	(Laugel *et al.*, 2009)
p.Arg637Serfs*34<br>p.Asp749Glufs*4	CS543VI	CS III	(Laugel *et al.*, 2009)
p.Glu182Asnfs*4<br>p.Glu379*	CS393VI	CS III	(Laugel *et al.*, 2009)
p.Ser1240_Val1260delinsIle<br>p.Arg735*	CS823VI	CS III	(Laugel *et al.*, 2009)
p.Lys1203fs<br>p.Met867Thrfs*14	CS1ABR	CS II	(Mallery *et al.*, 1998)
p.Tyr1179Ilefs*22<br>p.Phe665_Gln723del	CS2BE	?	(Mallery *et al.*, 1998)
p.Arg735*<br>p.Arg453*	25627	CS I	(Mallery *et al.*, 1998)
p.Gln184*<br>p.Arg670Trp	CS4BR	CS I	(Mallery *et al.*, 1998)
p.Arg670Trp<br>p.Pro1042Leu	CS2BI	CS I	(Mallery *et al.*, 1998)
p.Ser142Asnfs*4	?	?	(Shehata *et al.*, 2014)
p.Lys337*<br>p.Tyr834Cysfs*25	CS1AN	CS I	(Troelstra *et al.*, 1992)

p.(Glu215*)<br>p.(Ser1259*)	?	?	(Wu *et al.,* 2016)
p.Asp532Gly<br>p.Leu536Trp	?	CS I	(Yu *et al.,* 2014)

- Question mark (?) is used to refer to missing informations and uncertainties.
- The mutations nomenclature was described following the HGVS recommendations (Dunnen *et al.,* 2006).
- * is used to indicate a stop codon.

References

Calmels N, Greff G, Obringer C, Kempf N, Gasnier C, Tarabeux J, Miguet M, Baujat G, Bessis D, Bretones P *et al.* (2016) Uncommon nucleotide excision repair phenotypes revealed by targeted high- throughput sequencing. Orphanet J Rare Dis 11:26.

Calmels N, Botta E, Jia N, Fawcett H, Nardo T, Nakazawa Y, Lanzafame M, Moriwaki S, Sugita K, Kubota M *et al.* (2018) Functional and clinical relevance of novel mutations in a large cohort of patients with Cockayne syndrome. J Med Genet 55:329-343.

Chebly A, Corbani S, Ghoch JA, Mehawej C, Megarbane A and Chouery E (2018) First molecular study in Lebanese patients with Cockayne syndrome and report of a novel mutation in ERCC8 gene. BMC Med Genet 19:6–12.

Colella S, Nardo T, Mallery D, Borrone C, Ricci R, Ruffa G, Lehmann AR and Stefanini M (1999) Alterations in the CSB gene in three Italian patients with the severe form of Cockayne syndrome (CS) but without clinical photosensitivity. Hum Mol Genet 8:935–941.

Colella S, Nardo T, Botta E, Lehmann AR and Stefanini M (2000) Identical mutations in the CSB gene associated with either Cockayne syndrome or the DeSanctis-Cacchione variant of Xeroderma pigmentosum. Hum Mol Genet 9:1171–1175.

Falik-Zaccai TC, Laskar M, Kfir N, Nasser W, Slor H and Khayat M (2008) Cockayne syndrome type II in a Druze isolate in Northern Israel in association with an insertion mutation in ERCC6. Am J Med Genet 146:1423–1429.

Hashimoto S, Suga T, Kudo E, Ihn H, Uchino M and Tateishi S (2008) Adult-onset neurological degeneration in a patient with Cockayne syndrome and a null mutation in the CSB Gene. J Invest Dermatol 128:1597–1599.

He C, Sun M, Wang G, Yang Y, Yao L and Wu Y (2017) Two novel mutations in ERCC6 cause Cockayne syndrome B in a Chinese family. Mol Med Reports 15:3957–3962.

Horibata K, Iwamoto Y, Kuraoka I, Jaspers NG, Kurimasa A, Oshimura M, Ichihashi M and Tanaka K (2004) Complete absence of Cockayne syndrome group B gene product gives rise to UV-sensitive syndrome but not Cockayne syndrome. Proc Natl Acad Sci U S A 101:15410–15415.

Jaakkola E, Mustonen A, Olsen P, Miettinen S, Savuoja T, Raams A, Jaspers NG, Shao H, Wu BL and Ignatius J (2010) ERCC6 founder mutation identified in Finnish patients with COFS syndrome. Clin Genet 78:541–547.

Kou Y, Shboul M, Wang Z, Shersheer Q, Lyu Z, Liu P, Zhao X and Tian J (2018) Novel frame shift mutation in ERCC6 leads to a severe form of Cockayne syndrome with postnatal growth failure and early death: A case report and brief literature review. Medicine (Baltimore) 97:e11636

Laugel V, Dalloz C, Tobias ES, Tolmie JL, Martin-Coignard D, Drouin-Garraud V, Valayannopoulos V, Sarasin A and Dollfus H (2008) Cerebro-oculo-facio-skeletal syndrome: Three additional cases with CSB mutations, new diagnostic criteria and an approach to investigation. J Med Genet 45:564–571.

Laugel V, Dalloz AC, Durand M, Sauvanaud F, Kristensen U, Vincent MC, Pasquier L, Odent S, Gener B, Tobias ES *et al.* (2009) Mutation update for the CSB/ERCC6 and CSA/ERCC8 genes involved in Cockayne syndrome. Hum Mutat 31:113–126.

Luo Y, Ling Y, Chen J, Xu X, Chen C, Leng F, Cheng J, Chen M and Lu Z (2014) A new mutation in the CSB gene in a Chinese patient with mild Cockayne syndrome. Clin Case Reports 2:33–36.

Mallery DL, Tanganelli B, Colella S, Steingrimsdottir H, van Gool AJ, Troelstra C, Stefanini M and Lehmann AR (1998) Molecular analysis of mutations in the CSB (ERCC6) gene in patients with Cockayne syndrome. Am J Hum Genet 62:77–85.

Meira LB, Graham JM, Greenberg CR, Busch DB, Doughty AT, Ziffer DW, Coleman DM, Savre-Train I and Friedberg EC (2000) Manitoba aboriginal kindred with original cerebro-oculo-facio-skeletal syndrome has a mutation in the Cockayne syndrome Group B (CSB) Gene. Am J Hum Genet 66:1221–1228.

Shehata L, Simeonov DR, Raams A, Wolfe L, Vanderver A, Tifft CJ, He M, Jaspers NGJ and Gahl WA (2014) ERCC6 dysfunction presenting as progressive neurological decline with brain hypomyelination. Am J Hum Genet 164:2892–2900.

Swartz JM, Akinci A, Andrew S, Sigirci A, Hirschhown JN, Rosenfeld RG, Dauber A and Hwa V (2015) A novel ERCC6 splicing variant associated with a mild Cockayne syndrome phenotype. Horm Res Paediatr 27:320–331.

Troelstra C, van Gool A, de Wit J, Vermeulen W, Bootsma D and Hoeijmakers JHJ (1992) ERCC6, a member of a subfamily of putative helicases, is involved in Cockayne's syndrome and preferential repair of active genes. Cell 71:939–953.

Wilson BT, Lochan A, Stark Z and Sutton RE (2016) Novel missense mutations in a conserved loop between ERCC6 (CSB) helicase motifs V and VI : Insights into Cockayne syndrome. Am J Med Genet Part A 170:773–776.

Wu Y, Zheng Y, Yan X, Huang Y, Jiang Y and Li H (2016) Ocular findings in a patient with Cockayne syndrome with two mutations in the ERCC6 gene. Ophtalmic Genet 38:175–177.

Yu S, Chen L, Ye L, Fei L, Tang W and Tian Y (2014) Identification of two missense mutations of ERCC6 in three chinese sisters with Cockayne syndrome by Whole Exome Sequencing. PloS One 9:1–11.

Zhang H, Gao J, Ye J, Gong Z and Gu X (2011) Maternal origin of a de novo microdeletion spanning the ERCC6 gene in a classic form of the Cockayne syndrome. Eur J Med Genet 54:e389–e393.
