# Supplementary material for: Cockayne Syndrome: The many challenges and approaches to understand a multifaceted disease
Source: Genet Mol Biol. 2020 May 20;43(1 Suppl 1):e20190085. doi: 10.1590/1678-4685-GMB-2019-0085 (PMC7250278; doi:10.1590/1678-4685-GMB-2019-0085)
Supplement: Table S2 [file 1415-4757-GMB-43-1-s1-e20190085-suppl2.pdf]

Supplementary Material to "Cockayne Syndrome: The many challenges and approaches to understand a multifaceted disease"

Table S2 - Homozygous and heterozygous *ERCC8 (CSA)* mutations and their effects on patients phenotype.

CSA - Homozygous			
Mutation	Patient code	CS type	Reference
p.Asp266Gly	3_1<br>3_2	CS I	(Bertola *et al.*, 2006)
p.Glu55Lysfs*13	CS30PV	CS I	(Biancheri 2014)
p.(*His244Tyr*)	CS276ST	CS III ?	(Calmels *et al.*, 2016)
p.*Thr265Pro*	CS260ST	CS III ?	(Calmels *et al.*, 2016)
p.Glu13*	CS15PV<br>CS2JE	CS I	(Calmels *et al.*, 2018)
p.(Arg268*)	CS240ST	CS I/II	(Calmels *et al.*, 2018)
p.*Leu271Pro*	CS3BR	CS II	(Calmels *et al.*, 2018)
p.[Tyr322*;Val282_Gln347del; Val282_Glu374del]	CS9IAF	CS I	(Calmels *et al.*, 2018)
p.Val27_Arg92del	CS1LE	CS I	(Calmels *et al.*, 2018)
p.Leu251Tyrfs*18	CS172ST	CS II	(Calmels *et al.*, 2018)
p.Ala240Glyfs*8	CS7PV	CS II	(Calmels *et al.*, 2018)
p.Tyr200Lysfs*12	CS1GLO	CS I	(Calmels *et al.*, 2018)
p.Asn75Glnfs*9	CS4PV	CS II	(Calmels *et al.*, 2018)
p.(Gln106*)	CS263ST	CS I	(Calmels *et al.*, 2018)
p.Thr134Leufs*13	CS261ST	CS I/II	(Calmels *et al.*, 2018)
p.Tyr322*	?	CS I	(Chebly *et al.*, 2018)

p.*Tyr350Cys*	GM02965	CS III	(Kennedy *et al.*, 1980)
p.Gly184Aspfs28*	CSA10NO	CS I	(Kleppa *et al.*, 2007)
p.Asp266Gly	08STR2	CS I	(Laugel *et al.*, 2009)
p.Ala207_Ser209del	CS794VI	CS I	(Laugel *et al.*, 2009)
p.Leu202Ser	08STR3	CS I	(Laugel *et al.*, 2009)
p.Ala160Thr	CS852VI	CS III	(Laugel *et al.*, 2009)
p.Gly184Aspfs28*	CS7BI<br>CS7BI2<br>CS7BI3	CS I	(Laugel *et al.*, 2009)
p.Tyr322*	CS886VI<br>CS887VI	CS I	(Laugel *et al.*, 2009)
p.Tyr200Lysfs*12	CS24PV<br>CS417VI	?	(Laugel *et al.*, 2009)
p.Glu13*	CS218ST	CS I	(Laugel *et al.*, 2013)
p.Trp361Cys	UVS^{S}VI	UVSS	(Nardo *et al.*, 2009)
p.Thr134Leufs*13	CS133NY	CSI/III	(Rapin *et al.*, 2009)
p.Glu374Asp	?	CS I	(Taghdihi 2017)
***CSA* - Heterozygous**			
Mutation	Patient code	CS type	Reference
p.Val105Thrfs*6<br>p.Thr204Lys	?	CS I	(Bertola *et al.*, 2006)
p.Glu13*<br>p.Ala205Pro	AGO7075	CSI	(Cao *et al.*, 2004)
p.Thr134Leufs*13<br>p.Thr134Valfs*7	CS6PV	CS I	(Calmels *et al.*, 2018)
p.Asp199Metfs*14<br>p.Ser220*	CS16PV	CS I	(Calmels *et al.*, 2018)
p.Ala207_Ser209del<br>p.0	CS1JE	CS I	(Calmels *et al.*, 2018)

p.Pro95Leufs*30<br>p.Val161Serfs*5	CS9LO	CS I/II	(Calmels *et al.*, 2018)
p.Ser119Leu<br>p.Ala207_Ser209del	CS040ST	CS I	(Calmels *et al.*, 2016)
p.Tyr100*<br>p.Thr134Valfs*7	CS11PV	CS I	(Conte *et al.*, 2009)
p.Leu132fs*137<br>p.Ala240fs*243	?	CS I	(Gu *et al.*, 2017)
p.Trp194Cys<br>p.Thr24Asnfs*11	CS655VI	CS I	(Laugel *et al.*, 2009)
p.Asp266Gly<br>p.Ala240Glyfs*8	CS861VI	CS I	(Laugel *et al.*, 2009)
p.Asp93Leufs*26<br>p.Gln106Pro	CS2SE	?	(Ren *et al.*, 2003)
p.Ala160Val<br>p.Glu13*	CS3BE	CS I	(Ridley *et al.*, 2005)
p.Ala207_Ser209<br>p.Asp93Leufs*26	?	CS I	(Xie *et al.*, 2017)

- Question mark (?) is used to refer to missing informations and uncertainties.
- The mutations nomenclature was described following the HGVS recommendations (Dunnen *et al.*, 2006).
- * is used to indicate a stop codon.

References

Bertola DR, Cao H, Albano LM, Oliveira DP, Kok F, Marques-Dias MJ, Kim CA and Hegele RA (2006) Cockayne syndrome type A: Novel mutations in eight typical patients. J Hum Genet 51:701–705.

Biancheri R, Mancardi MM, Celle ME, Nardo T and Morana G (2014) Cranial nerve and cauda equina contrast enhancement in Cockayne syndrome. Neurology 83:1581.

Calmels N, Greff G, Obringer C, Kempf N, Gasnier C, Tarabeux J, Miguet M, Baujat G, Bessis D, Bretones P *et al.* (2016) Uncommon nucleotide excision repair phenotypes revealed by targeted high- throughput sequencing. Orphanet J Rare Dis 11:26.

Calmels N, Botta E, Jia N, Fawcett H, Nardo T, Nakazawa Y, Lanzafame M, Moriwaki S, Sugita K, Kubota M *et al.* (2018) Functional and clinical relevance of novel mutations in a large cohort of patients with Cockayne syndrome. J Med Genet 55:329-343.

Cao H, Williams C, Carter M and Hegele RA (2004) CKN1 (MIM 216400): Mutations in Cockayne syndrome type A and a new common polymorphism. J Hum Genet 49:61–63.

Chebly A, Corbani S, Ghoch JA, Mehawej C, Megarbane A and Chouery E (2018) First molecular study in Lebanese patients with Cockayne syndrome and report of a novel mutation in ERCC8 gene. BMC Med Genet 19:6–12.

Conte C, D'Apice MR, Botta A, Sangiuolo F and Novelli G (2009) Prenatal diagnosis of Cockayne syndrome type A based on the identification of two novel mutations in the ERCC8 gene. Genet Test Mol Biomarkers 13:127–131.

Gu Y, Bhatta AK, Du X, Shao M, Keyal U and Hua Y (2017) Identification of one novel complex delins mutation and one recurrent mutation of ERCC8 gene in a Chinese family with Cockayne Syndrome A. J Eur Acad Dermatology Venereol 31:e394–e395.

Kennedy RM, Rowe VD and Kepes JJ (1980) Cockayne syndrome: An atypical case. Neurology 30:1268–1272.

Kleppa L, Kanavin J, Klungland A and Strømme P (2007) A novel splice site mutation in the Cockayne syndrome group A gene in two siblings with Cockayne syndrome. Neuroscience 145:1397–1406.

Laugel V, Dalloz AC, Durand M, Sauvanaud F, Kristensen U, Vincent MC, Pasquier L, Odent S, Gener B, Tobias ES *et al.* (2009) Mutation update for the CSB/ERCC6 and CSA/ERCC8 genes involved in Cockayne syndrome. Hum Mutat 31:113–126.

Laugel V (2013) Cockayne syndrome: The expanding clinical and mutational spectrum. Mech Ageing Dev 134:161–170.

Nardo T, Oneda R, Spivak G, Vaz B, Mortier L, Thomas P, Orioli D, Laugel V, Stary A, Hanawalt PC *et al.* (2009) A UV-sensitive syndrome patient with a specific CSA mutation reveals separable roles for CSA in response to UV and oxidative DNA damage. Proc Natl Acad Sci U S A 106:6209–6214.

Rapin I, Weidenheim K, Lindenbaum Y, Rosenbaum P, Merchant SN, Krishna S and Dickson DW (2009) Cockayne Syndrome in adults: Review with clinical and pathologic study of a new case. J Child Neurol 21:991–1006.

Ren Y, Saijo M, Nakatsu Y, Nakai H, Yamaizumi M and Tanaka K (2003) Three novel mutations responsible for Cockayne syndrome group A. Genes Genet Syst 78:93–102.

Ridley AJ, Colley J, Wynford-Thomas D and Jones CJ (2005) Characterisation of novel mutations in Cockayne syndrome type A and Xeroderma pigmentosum group C subjects. J Hum Genet 50:151–154.

Taghdiri M, Dastsooz H, Fardaei M, Mohammadi S, Fard MAF and Faghihi MA (2017) A Novel Mutation in ERCC8 Gene Causing Cockayne Syndrome. Front Pediatr 5:169.

Xie H, Li X, Peng J, Chen Q, Gao Z, Song X and Li W (2017) A complex intragenic rearrangement of ERCC8 in Chinese siblings with Cockayne syndrome. Sci Rep 7:44271.
